# Challenges faced with the implementation of Web-Based Data Query Systems for population health: development of a questionnaire based on expert consensus

**DOI:** 10.1186/s40814-018-0307-3

**Published:** 2018-06-14

**Authors:** Manik Ahuja, Robert Aseltine, Nicholas Warren, Susan Reisine, Pam Holtzclaw Williams, Andy Cislo

**Affiliations:** 1 Brown School of Social Work, Washington University, St. Louis, MO 63110 USA; 2 Center for Population Health, University of Connecticut Health Center, Farmington, CT 06032 USA; 3 Division of Occupational and Environmental Medicine, University of Connecticut Health Center, Farmington, CT 06032 USA; 4 Division of Behavioral Sciences and Community Health, University of Connecticut Health Center, Farmington, CT 06032 USA; 5 Department of Nursing, University of Arkansas Medical Sciences (UAMS), Little Rock, AR 72205 USA

**Keywords:** WDQS, Web-Based Data Query Systems, State public health query systems, Public health, Health surveillance, Public health query systems, “Disseminated” and “public health”, State public health aggregate level data, “State agency” and “public health”, “Data” and “public health agencies”

## Abstract

**Background:**

State health agencies (SHA) and local health agencies (LHA) face several challenges with the dissemination of local health data using Web-Based Data Query Systems (WDQS). To help guide future research, this study aimed to utilize expert consensus to identify the most relevant items that contribute to these challenges.

**Methods:**

A total of 17 researchers and public health professionals agreed to participate in a three-round Delphi process. In round 1, four topics were represented on a 42-item questionnaire using a 5-point Likert scale, along with free-text responses. Free-text responses were analyzed leading to a series of items for a second Delphi round. Participants were given an opportunity to revise results in round 3 for items that did not meet consensus in round 1 or round 2. Consensus on expert opinions was defined at interquartile range (IQR) ≤ 1.

**Results:**

The experts reached consensus on a total of 21 (50%) of the 42 items presented in the initial questionnaire. Eleven of the 15 (73%) of the items extracted from the free-text responses met consensus. Items in consensus from this pilot study were used to develop an instrument for a broader survey across Behavioral Risk Factor Surveillance System (BRFSS) coordinators across all 50 US states.

**Conclusion:**

Experts confirmed that software development costs, inadequate human resources, data sharing gaps, a lack of political support, and poor data quality contribute significantly to challenges in their data implementation. The findings from this pilot study inform us of items of public health significance that will help guide future research.

## Background

Local health data can be a powerful vehicle for improving the health of a community [1]. When aggregated, local health data helps monitor the incidence, trends, and patterns and disease in a given population [2]. There is strong evidence that the availability of high-quality population level health data at the local level can lead to targeted interventions, impact public policy decisions [3], reduce health disparities, and improve health care delivery systems [4]. The growth of the Internet over the last 25 years has made it possible for state agencies to easily share their health data online. One popular method to disseminate health data are Web-Based Data Query Systems (WDQS), which were first implemented in the late 1990s. WDQS are interactive and are customizable, as users are able to pre-select variables of interest [5]. Despite the advantages of WDQS and advancements in information technology, implementation has been limited. States and local health agencies face key challenges including high cost, data sharing, IT infrastructure, and usability challenges in their dissemination. This paper reports on the relevance of barriers identified in the literature, from the perspective of a panel of experts. The results of this pilot study helped formulate an instrument that was administered to Behavioral Risk Factor Surveillance System (BRFSS) coordinators across all 50 US states.

### Cost challenges

WDQS are expensive to design, develop, and maintain. To receive funding for health IT-related projects, political support or “buy in” from relevant stakeholders is necessary [6, 7]. State budgets are generally year to year, which prevents long-term planning for implementation of systems [8–10]. It is critical to secure adequate resources in the long term to maintain systems, ensure data are current, and to keep systems operable. The high cost of hardware, software, staffing, and project management are barriers that contribute to cost challenges.

### Data sharing challenges

Data sharing remains a significant challenge for state and local health departments [11]. One of the most difficult challenges is access to complete and usable population health data [11]. Data sharing is necessary in order to have a complete picture of a population’s health at the local level [12]. Barriers to data sharing are caused by both technical and non-technical factors [12]. Examples of technical factors include missing primary identifiers, disaggregation of indicators, incompatibility of systems, and the inability to identify data elements. Examples of non-technical factors include reluctance of agencies or organizations/hospitals to release data, institutional review board issues, as well as legal and political issues [4, 12–14].

#### IT infrastructure and usability challenges

IT infrastructure refers to the composite hardware, software, network resources, and data storage for IT operations. Adequate data storage is vital as health datasets can consume large amounts of data storage space. Usability problems have also been reported, as systems are often difficult to navigate and use, and data are missing or incomplete [1]. Usability includes the functionality, the ability to retrieve data, and the usefulness of these data. Usability is evaluated by user-computer interactions and by the degree of successful completion of an intended task [15]. Poor usability may lead to poor perception among users, making them less likely to return as future users [16].

### The Delphi study

This study aimed to ascertain the importance of barriers that organizations face in the dissemination of local health data. The Delphi method was chosen, due to its suitability for areas of inquiry where incomplete knowledge exists [17]. It is an iterative, multi-stage, group-oriented process that involves a series of structured questionnaires [17], designed to transform opinion into group consensus. Using experts, this method seeks to gain a group consensus on a specific topic from individuals as consensus is defined as a “general agreement of a substantial majority” [10]. The study involves a series of questionnaires administered to the experts, using multiple rounds. We chose to use a controlled feedback method known as “quasi-anonymous feedback,” in which names of the participants are known only to the researcher and not to others in the group [18]. It is known as “quasi” anonymous because complete anonymity cannot be guaranteed, as the researcher knows the name of the panel members and their responses. Anonymity among the participants eliminates problems with bias and peer influence and reduces the effect of dominant individuals [19]. The study was designed to answer the following question: which topics should be prioritized for future decision-making regarding best practices of WDQS implementation?

## Methods

### Delphi process

#### Selection of panel members and recruitment

A purposive sample of 17 experts agreed to participate in this study. Purposive sampling is a non-probability sampling that uses the judgement of the researcher to recruit participants. Purposive samples are often used in Delphi studies [20]. Since expert opinion is sought, a purposive sample is necessary when people are selected not to represent the general population, but rather are selected for their expert ability to answer specific research questions [21]. It is recommended that participants have either published articles, taught courses about the topic, or a professional role related to the area [22]. In the current study, panel members had to have met at least *one* of the following criteria: (1) have published relevant literature in peer-reviewed scholarly journals or (2) a significant portion of their job responsibility involves the dissemination of local health data using WDQS. We searched online for authors with relevant publications or practitioners, derived contact information, and contacted the authors by email. Potential participants working with the dissemination of WDQS were identified through multiple channels. We searched on state and local health department websites along with the *Naphsis* website, an organization whose mission is to provide health information to improve the public’s health. We searched the Department of Health website from states that have previously implemented WDQS and attempted to find knowledgeable staff. All participants were contacted through email.

#### Ethical consideration

The Delphi study participants were informed that their participation in the study was entirely voluntary, and they implicitly consented to participate by completing the questionnaire. They were also informed that their results were confidential as well as their names. Their names are not provided to other participants in the study. The approval for the study was received from the Human Subjects Protection Office at the University of Connecticut Health Center on July 3, 2014, as it was determined that the project was exempt from IRB review.

#### Procedure

**Fig. 1:**
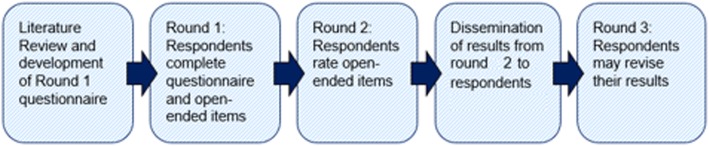
Schematic of three Delphi rounds

We conducted a three-round Delphi study (Fig. 1). We selected a three-round study, as it is efficient and typical of most Delphi studies [23]. All rounds of the Delphi study were conducted through email, and each questionnaire was administered in a Microsoft Word format. Participants were given approximately 1 week to complete each round, and a reminder email was sent following the deadline. To ensure strong retention of expert involvement, the study was set at three rounds. It is known that having a planned number of rounds is an indicator of good quality in designing a Delphi study [24].

#### Overview

In the first round, we administered a 42-item questionnaire along with an open-ended response section. In the open-response section, participants were able to list additional items of importance that were not included in the questionnaire. In the second round, we administered a questionnaire based on a filtered list of open-ended responses from round 1. In round 3, participants were presented a list of items that did not meet consensus in round 1 and round 2. They were given an opportunity to revise their results in order to come to consensus with the group.

#### Round 1: questionnaire

A four-topic, 42-item questionnaire was administered. There were four categories of topics including cost, data collection, IT infrastructure, and usability. Within each topic were a series of items that were relevant to that topic. Participants were asked to rate the importance they would give to each item on 5-point Likert scale (Unimportant—1; Little importance—2; Moderate importance—3; Important—4; Very important—5) or N/A (Not applicable).

Results from the 42-item questionnaire were compiled in Microsoft Excel and then imported to SPSS v 19.0 for analysis. The mean, median, and interquartile range (IQR) were calculated for each item. The IQR is a measure of statistical dispersion, being equal to the difference between the mean score of the upper and lower quartiles, and thus consists of the middle 50% of the observations [25]. An IQR of less than 1 indicates that more than 50% of all responses fall within 1 point on the scale [26]. It is a frequently used measure in Delphi studies, and it is generally accepted as an objective and rigorous way of determining consensus [27]. Items with an IQR of 1 or less can be considered to demonstrate good consensus on a 5-point Likert scale. Items that met consensus in round 1 or round 2 achieved consensus and no further data need to be collected for that item. Items that do not meet consensus were carried over to round 3.

#### Round 1: open-text response section

In the optional open-text response sections, participants listed key barriers they face but were not captured in the initial questionnaire. A cumulative list of all open-text items was analyzed using Microsoft Excel. *Qualitative content analysis* was used to identify and interpret themes in the qualitative material. Qualitative content analysis is a technique for systematic text analysis, which uses themes to identify qualitative responses. Researchers regard content analysis as a flexible method for analyzing open-ended, qualitative, or text-based responses [28]. As the sample size was relatively small, thus data were manually sorted by project staff, and common themes were formed from these data.

#### Second round questionnaire

A compiled list of participant-generated items from round 1 was distributed to participants. Participants rated the importance of each item, as previously described. These responses from the round 2 questionnaire were entered into Microsoft Excel and then imported to SPSS v 19.0 for analysis. Items that achieved an IQR of ≤ 1 met consensus, and items with an IQR > 1 were carried over to round 3.

#### Third round

In the final round of the Delphi process, respondents were provided a list and median score of items from rounds 1 and 2 that did not meet consensus. Participants were given an opportunity to revise their estimates from rounds 1 and 2. We calculated the mean, median, and IQR after round 3. Items with an IQR ≤ 1 met consensus. Items with an IQR > 1 were not in consensus. Participants also were given an opportunity to qualitatively provide a reason if they chose not to revise an estimate.

## Results

### Participants

Overall, 17 experts agreed to participate in the study. Of the 17 participants that agreed to participate, 15 (88%) of them submitted completed round 1 responses. One further participant dropped out of the study after round 1; 14 participants (82%) completed both round 2 and round 3. Of the 14 experts who completed the survey, eight had published literature on the development, evaluation, or the dissemination of WDQS. Those who published literature included researchers, college/university professors, and others in academia with expertise. The remaining six experts include public health professionals. This group consisted of experts such as epidemiologists, health directors, and other key informants in the public health community who were involved in the dissemination of WDQS.

### Summary of rounds

#### Round 1 and round 2

The summary for each round is presented in Table 1. This table presents an overview of the scoring for each round. Items that met consensus in rounds 1 and 3 are presented in Table 2. Items that did not meet consensus are presented in Table 3. In round 1, 14 of the 42 (33%) of the items met consensus, and 28 items were carried over (67%) to round 3 because consensus was not reached. In round 2, 10 out of the 15 open-ended items (67%) met consensus and were retained. The five items (33%) that did not meet consensus in round 2 were presented in the round 3 for an opportunity to revise.

**Table 1:** Overall consensus from round 1 and round 3 for quantitative items

		Round 1 consensus		Round 3 consensus	
Topic	Total no. of items	Items in consensus	Percent consensus	Items in consensus (*n*)	Percent consensus
Cost	13	5	38	7	54
Data collection	13	4	31	6	46
IT infrastructure	6	2	33	2	33
Usability	10	3	30	6	60

**Table 2:** Items that met consensus using a 5-point Likert scale

Topic	Item	Mean	Median	IQR	Number	Round consensus was achieved
Cost	Cost to have adequate state agency (public health staff)	4.33	4	1	15	Round 1
	Cost of system design/software development	4.14	4	1	15	Round 1
	Cost to have adequate staff/headcount for IT staff (internal)	4.07	4	1	15	Round 1
	Cost of IT technical support for state agency staff	3.46	3.5	1	14	Round 3
	Cost of technical support to end users	3.13	3.5	1	14	Round 3
	Cost of servers/hosting applications	3.00	3	0.25	15	Round 1
	Cost of data storage	2.40	2	0.5	15	Round 1
Data collection	Challenges in acquiring data that are useful and meaningful	4.63	5	0.75	15	Round 1
	Challenges in acquiring data that have been requested by relevant stakeholders/end users	4.42	5	1	15	Round 1
	Challenges in acquiring data from multiple data sources across the state	4.23	4	1	15	Round 1
	Challenges in working with private hospitals and clinics to release data	4.21	4.5	1	14	Round 3
	Collecting data in a timely manner	4.21	4	1	15	Round 1
	Challenges in working with public hospitals and clinics to release data	4.01	4	1	14	Round 3
IT infrastructure	Challenges in collaboration with software developers and IT staff	3.57	4	1	15	Round 1
	Challenges in decision making on technology (open source, commercial, etc.	3.53	4	1	15	Round 1
Usability	Data are meaningful and is useful for the end user	4.40	5	1	15	Round 1
	Data are missing or incomplete for end user	3.80	4	1	14	Round 3
	Quality of user data output (Excel, csv, pdf, html etc.	3.71	4	0.75	15	Round 1
	Drill downs/data filers are difficult to understand	3.50	4	0.75	15	Round 1
	Website freezes up	3.33	3.5	1	14	Round 3
	Data do not go far back enough in time	3.21	3.5	1	15	Round 3
Qualitative items (participant generated	Evaluation of end users	4.57	5	1	14	Round 2
	Standardization of vocabulary	4.50	5	1	14	Round 2
	Providing context in a way which makes a “story” of the data	4.50	4.5	1	14	Round 2
	Hidden costs associated with development	4.42	5	1	14	Round 2
	A greater understand of how the consumer consumes the information	4.42	5	1	14	Round 2
	Need for “user centric” design	4.35	4.5	1	14	Round 2
	Helpdesk support for end users	4.28	4	1	14	Round 3
	Data from the private sector	4.07	4	1	14	Round 2
	Using existing public health surveillance systems and mandated hospital discharge reporting maintained by state department of health	3.72	4	0.5	14	Round 2
	Rigorous validation of data and statistical algorithm	3.20	3	0.25	14	Round 2
	Evaluation of end users	4.57	5	1	14	Round 2

Items with an IQR ≤ 1 met consensus

*Mean* mean score, *Median* median score, *IQR* interquartile range

**Table 3:** Items that did not meet consensus after round 3

Item	Mean	Median	IQR
Navigation and website buttons are clear, concise, and easy to understand	4.14	5	1.75
Challenges in having “buy in” from state governments	4.00	4	2
Challenges in linking across multiple data sources	4.00	4.5	2
Cost of system maintenance after deployment	3.71	4	1.75
Availability of IT support staff by email or phone for technical questions	3.71	3.5	2
Resources for improving and updating systems	3.57	4	1.75
WDQS links/URL’s do not work or links within website do not work	3.57	4	2
Challenges in having “buy in” from local governments	3.53	3.5	2.25
Cost of software testing and QA/QC control testing	3.47	3.5	1.75
Privacy issues with small cell counts with aggregate data	3.47	3	2
Reliability of systems	3.35	3	3
Systems are not compatible with end user web browser	3.35	1.5	3
Data types mismatch when importing data	2.97	3	3
Cost of commercial software	2.92	3	2.25
Time to process queries is long	2.92	3	2
Challenges in acquiring vital statistics	2.85	3	1.75

Items with an IQR > 1 did not meet consensus

*Mean* mean score, *Median* median score, *IQR* interquartile range

#### Final round

All items that met consensus are shown in Table 2. After participants had a chance to revise, consensus was reached in 21 of the 42 items (50%) that were originally presented in round 1. Fourteen of the 21 items met consensus in the initial round 1, while 7 of these items had met consensus after participants had an opportunity to revise their scores in round 3. Overall, 60% of the usability items presented met consensus, followed by cost (54%), data collection (46%), and IT infrastructure (33%). Of participant generated/open-text response items, overall, 11 of the 15 items (73%) met consensus. Ten items met consensus in round 1, while 1 additional item met consensus in round 3 after participants had an opportunity to revise their score.

### Results by topic

#### Cost

Cost of adequate public health staff (mean = 4.33, IQR = 1), the cost of system development (mean = 4.14; IQR = 1), and IT staff (mean = 4.07; IQR = 1) met consensus and were rated highest. The cost of IT technical support for state agency staff (mean = 3.46; IQR = 1), IT technical support for end users (mean = 3.13, IQR = 1), hardware/servers (mean = 3.00; IQE = 0.25), and cost of data storage (mean = 2.40; IQR = 0.5) met consensus but were rated lower.

#### Data collection

Acquiring data that are useful and meaningful (mean = 4.63; IQR = 0.75) was rated highest. Other items that met consensus include acquiring data that have been requested by end users (mean = 4.42; IQR = 1), acquiring data from multiple sources (mean = 4.43; IQR = 1), collecting data in a timely manner (mean = 4.21; IQR = 1), and collecting data from hospitals and clinics.

#### IT infrastructure

IT infrastructure barriers were rated lower in comparison to the other domains. Two items achieved consensus, including collaboration with software developers to ensure systems needs are met (mean = 3.57; IQR = 1) and challenges in decision-making on software options (mean = 3.53; IQR = 1).

#### Usability

The most significant barriers to usability pertained to accessibility of data. Participants rated the following topics highest: meaningful and useful data (mean = 4.40; IQR = 1), missing or incomplete data (mean = 3.80; IQR = 1), quality of data output (mean = 3.71; IQR = 0.75) Navigational issues including difficulty in interpreting drilldowns (mean = 3.5; IQR = 0.75) and bandwidth issues including websites that freeze up (mean = 3.33, IQR = 1) met consensus but were rated slightly lower.

## Discussion

Access to high-quality data is paramount for local health departments, researchers, policymakers, and other key stakeholders who are involved with the health systems. The results from this pilot study have found that cost and data sharing are the two majors of the most significant barriers that state agencies face with their dissemination. One way to maximize resources is for state agencies to collaborate with other states. This includes software development, strategy, and forming partnerships with other states. Through collaboration, states can share their software costs and reduce the level of planning, design, and development costs. Another option is for states to use open-source technologies, which are of little or no cost. In addition, through open-source, software coding is easily adaptable and can be available through the public domain.

Acquiring data from private and public hospitals along with other sources across a state was deemed a major barrier in this study. This study found that state agencies are faced with challenges in accessing data from multiple sources. According to the Centers for Disease Control and Prevention (CDC), one of the major challenges is to find effective ways of combining multiple sources of complex data [11]. Linking data from multiple sources may help provide information regarding social determinants of disease or key demographic information, which helps give us a more complete description of an affected population [29, 30]. One mechanism to overcome challenges with data sharing is to increase efforts towards open data, which some states have recently adopted. Open data is a repository of data that can be freely used and redistributed by anyone [31]. Open data creates opportunities for exchange, in which organizations can share their own data, and use other data that are available [32].

Experts from this study agreed that there is a lack of standardization in vocabulary across systems, and users are having a difficult time interpreting data from one system to the other. As an example, one system may use “ethnicity” and “race” as one variable, while another system may use them as two separate variables. Another example is one state that may report “percent overweight,” while another state may report “percent obese.” There is a need for standardization and transparency across systems so researchers can rely on state-level population data and be able to analyze data across states in a meaningful way [6, 33].

To surprise, several items that were found to be significant in our literature search did not meet consensus. Items such as buy-in from local and state governments, website usability, resources for maintenance and updating systems, system reliability, and data privacy issues did not meet consensus, even after participants were given an opportunity to revise. For example, issues around data privacy and security have been highly emphasized in academic literature. However, from the perspective of our experts, having access to data that are meaningful and useful was of higher significance than issues around data privacy issues. One possible explanation are the recent technological advancements which have led to more robust and improved data protection software and hardware for health data [34]. However, despite the advancements in technology, non-technical problems such as data sharing and costs for system development remain a top priority for state and local health agencies.

Overall, results from this pilot study provided us the requisite knowledge for a subsequent study administered to Behavior Risk Factor Surveillance Systems (BRFSS) coordinators across all 50 US states in 2015. Items that were in consensus derived from this pilot study were instrumental in developing our survey, as the results help guide the subsequent study.

## Conclusions

Past research highly emphasized technology-centered problems, such as usability, bandwidth, and slow computer processors, as barriers to implementation. However, information technology has substantially improved, making it easier to design and develop systems. It is imperative that greater investment into health data systems be made at the local and community level. Greater access to these data may help key stakeholders understand health problems of a community and subpopulations. This knowledge has the potential to facilitate prevention efforts and targeted interventions at the local and community levels.

## Abbreviations

LHA: Local Health Agency
SHA: State Health Agency
WDQS: Web-Based Data Query Systems
